# Deposits from giant floods in Gale crater and their implications for the climate of early Mars

**DOI:** 10.1038/s41598-020-75665-7

**Published:** 2020-11-05

**Authors:** E. Heydari, J. F. Schroeder, F. J. Calef, J. Van Beek, S. K. Rowland, T. J. Parker, A. G. Fairén

**Affiliations:** 1 Department of Physics, Atmospheric Sciences, and Geoscience, Jackson State University, 1400 Lynch Street, Jackson, MS 39217 USA; 2 Jet Propulsion Laboratory, California Institute of Technology, 4800 Oak Grove Drive, Pasadena, CA 91109 USA; 3 Department of Earth Sciences, University of Hawaii, Honolulu, HI 96822 USA; 4 Centro de Astrobiología (CSIC-INTA), Madrid, Spain; 5 Department of Astronomy, Cornell University, Ithaca, NY 14853 USA

**Keywords:** Climate sciences, Planetary science

## Abstract

This study reports in-situ sedimentologic evidence of giant floods in Gale crater, Mars, during the Noachian Period. Features indicative of floods are a series of symmetrical, 10 m-high gravel ridges that occur in the Hummocky Plains Unit (HPU). Their regular spacing, internal sedimentary structures, and bedload transport of fragments as large as 20 cm suggest that these ridges are antidunes: a type of sedimentary structure that forms under very strong flows. Their 150 m wavelength indicates that the north-flowing water that deposited them was at least 24 m deep and had a minimum velocity of 10 m/s. Floods waned rapidly, eroding antidune crests, and re-deposited removed sediments as patches on the up-flow limbs and trough areas between these ridges forming the Striated Unit (SU). Each patch of the SU is 50–200 m wide and long and consists of 5–10 m of south-dipping layers. The strike and dip of the SU layers mimic the attitude of the flank of the antidune on which they were deposited. The most likely mechanism that generated flood waters of this magnitude on a planet whose present-day average temperature is − 60 °C was the sudden heat produced by a large impact. The event vaporized frozen reservoirs of water and injected large amounts of CO_2_ and CH_4_ from their solid phases into the atmosphere. It temporarily interrupted a cold and dry climate and generated a warm and wet period. Torrential rainfall occurred planetwide some of which entered Gale crater and combined with water roaring down from Mt. Sharp to cause gigantic flash floods that deposited the SU and the HPU on Aeolis Palus. The warm and wet climate persisted even after the flooding ended, but its duration cannot be determined by our study.

## Introduction

**Figure 1:**
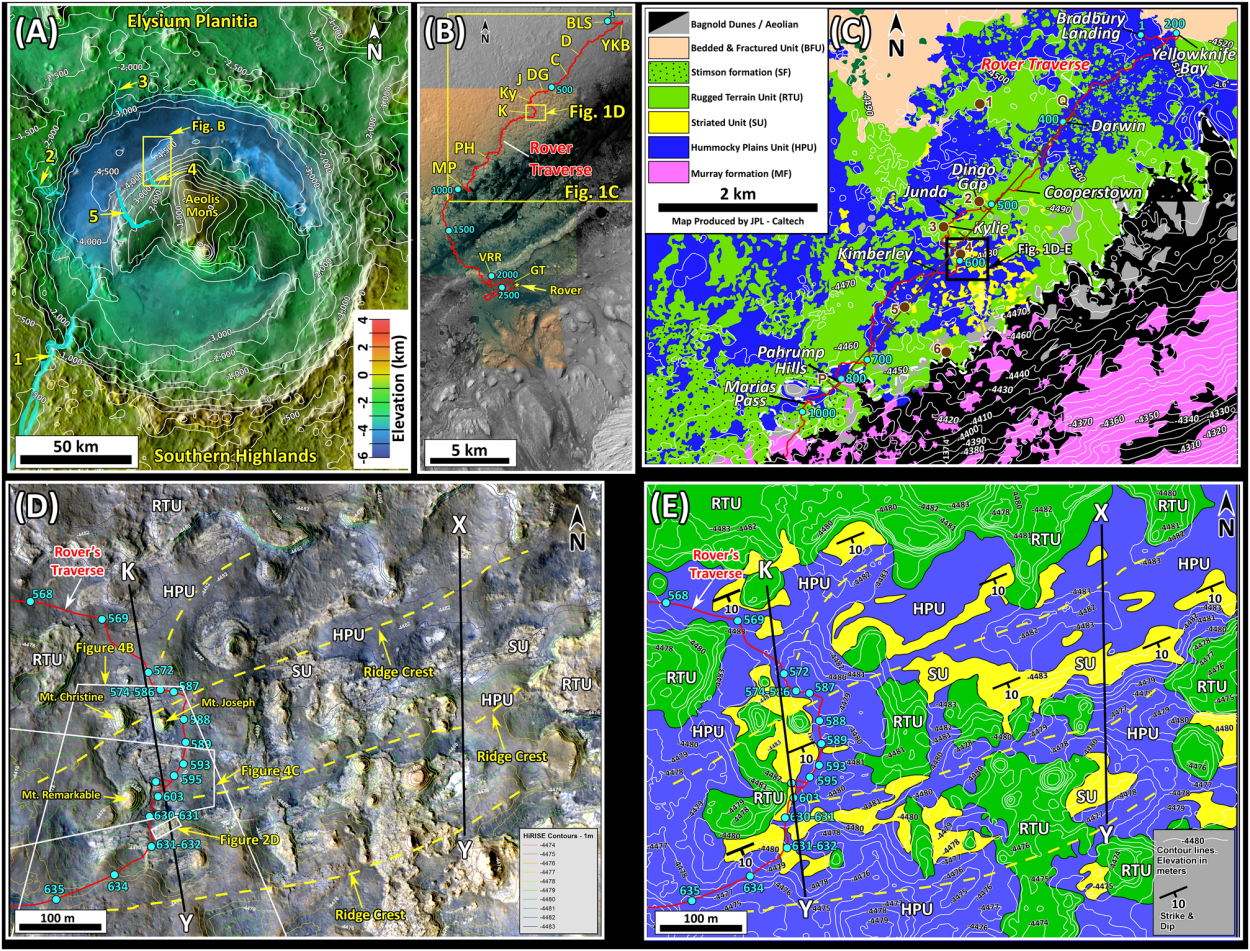
(**A**) Map shows physiographic features of Gale crater. White lines are topographic contours at 500 m intervals. The central mound (Aeolis Mons), informally known as Mt. Sharp, consists of 5 km of layered rocks. The crater floor is a ring-like low elevation area surrounding Mt. Sharp. The crater was once filled with sedimentary rocks but was subsequently excavated resulting in the present-day morphology^21–23^. The yellow rectangle is the area shown in (**B**). Major valleys and canyons are in cyan and include 1: Farah Vallis, 2: Dulce Vallis, 3: Peace Vallis, 4: Gediz Vallis, and 5: Grand Canyon of Gale crater (from^24^). The map was prepared in JMARS (Java Mission-Planning and Analysis for Remote Sensing) website v.4.0 (https://jmars.mars.asu.edu). JMARS is a cross-platform open source^25–28^ software that provides access to many Mars data products. Credit: Christensen et al.^25^, The map is a Thermal Emission Imaging System (THEMIS) Day IR (Infrared) image with elevation colors from Mars Orbiter Laser Altimeter (MOLA). Credits: Smith et al.^26^, Edwards et al.^27^, Hill and Christensen^28^, MOLA Science team, JPL, and NASA. (**B**). Map shows traverse path of the Curiosity rover. Large and small yellow squares are areas shown in (**C**) and (**D**), respectively. Solid cyan circles and cyan numbers indicate sol locations and sol numbers, respectively. *BLS* Bradbury Landing Site, *C* Cooperstown, *D* Darwin, *DG* Dingo Gap, *GT* Glen Torridon, *J* Junda, *K* Kimberley, *Ky* Kylie, *MP* Marias Pass, *PH* Pahrump Hills, VRR: Vera Ruben ridge. This figure was generated from High Resolution Imaging Science Experiment (HiRISE) base map for Mars Science Laboratory (https://bit.ly/MSL_Basemap). Credit: Calef and Parker^29^. (**C**) Geological map shows rock units exposed along part of the area investigated by the curiosity rover. The black square is the area shown in (**D**) and (**E**). White lines are topographic contour lines at 10 m intervals. Solid cyan circles and cyan numbers are sol locations and sol numbers, respectively. Brown circles and brow numbers are locations where the elevation of the SU is determined and projected to line P–Q and shown in a cross section on Fig. 5. (**D**) The HiRISE image of the Kimberly region shows characteristics of the Striated Unit (SU), the Rugged Terrain Unit (RTU), and the Hummocky Plains Unit (HPU). Layers of the SU strike N60° E and dip about 10° SE. Solid cyan circles and cyan numbers are sol locations and sol numbers, respectively. Yellow dashed lines are crests of ridges in the HPU. X–Y and K–Y show locations of geological cross sections in Fig. 3. Thin colored lines are topographic contours at 1 m intervals. The image was generated from HiRISE RGB color image ESP_027834_1755 (https://bit.ly/MSL_Basemap) with a standard deviation stretch using ArcGIS version 10.6. Credit: Calef and Parker^29^. (**E**) Geological map of the Kimberley region prepared from the HiRISE image in (**D**). White lines are topographic contours at one-meter interval. Solid cyan circles and cyan numbers are sol locations and sol numbers, respectively. X–Y and K–Y show locations of geological cross section in Fig. 3. Yellow dashed lines are the location of ridge crests which were also marked in (**D**).

Evidence of past catastrophic floods on Mars has been inferred from large channels, scour features, and mega ripples observed on orbital images^1–4^. The in-situ confirmation of these floods was first accomplished by examination of sedimentary deposits at the Mars Pathfinder landing site^5,6^. Here, sedimentary deposits are highly unsorted, with clasts ranging in size from sand to boulder^5,6^. Fragments are rounded to semi-rounded and some appear to be imbricated^5,6^. These sedimentological characteristics, as well as their location near the mouth of the Ares and Tiu valles, suggest that they were deposited by floods through these outflow channels^7^. The subsequent two decade-long investigations by the Spirit and the Opportunity rovers and the on-going observations by the Curiosity rover in Gale crater have discovered geological evidence of sedimentation by aeolian, fluvial, deltaic, and lacustrine processes^8–20^ but flood deposits have not yet been reported. In this investigation, we present sedimentological evidence for deposition by giant flash floods inside Gale crater, Mars, during the Noachian Period (Fig. 1).

Gale crater is located near the Martian equator (latitude: 5.3° S, longitude: 137.7° E) along the boundary that separates the Southern Highlands from the Northern Lowlands. It is the field site of the Mars Science Laboratory (MSL) rover Curiosity whose mission is to search for habitable environments on the red planet^30,31^. The proposed age of Gale crater ranges from the Early Noachian to the Early Hesperian depending on the method used^32–34^. However, a recent evaluation suggests that the crater was most likely formed during the Middle Noachian (3.95 to 3.75 billion years ago)^34^. After its formation, the crater was completely filled with sediments^21–23^. Its margins were subsequently excavated leading to the emergence of Mt. Sharp and the appearance of its modern morphology^21–23^. This geological history led to the extensive exposures of sedimentary rocks in Gale crater, making it an ideal place to study the evolution of early Mars^21–23^.

Gale crater has two physiographic provinces: Aeolis Mons, also known as Mt. Sharp, and the crater floor (Fig. 1A). Mt. Sharp is a crescent shaped mound near the center of the crater and consists of 5 km of sedimentary rocks (Fig. 1A). The crater floor is a ring-like depression that surrounds Mt. Sharp (Fig. 1A). Its northern portion is named Aeolis Palus (Fig. 1A). Many channels cut the rim of Gale crater (Fig. 1A). The largest one is Farah Vallis that is 3 km wide and 700 m deep (Fig. 1A). In addition, huge canyons are also carved into Mt. Sharp (Fig. 1A). One of them , called the Grand Canyon of Gale crater, is about 2 km wide and is 200–300 m deep (Fig. 1A).

The Curiosity rover landed on the Bradbury Landing Site on the northern margin of Aeolis Palus in August of 2012 (Figs. 1B, S1). It has been conducting detailed investigations along its path from the landing site toward Mt. Sharp (Figs. 1B, S1). Six rock units were identified before and after the landing of the rover (Fig. 1C). They are the Bedded and Fractured Unit (BFU), the Hummocky Plains Unit (HPU), the Striated Unit (SU), the Rugged Terrain Unit (RTU), the Murray formation (MF), and the Stimson formation (SF). Each has specific orbital attributes and distinct in-situ lithological and sedimentological characteristics^11,12^.

Our goal is to present sedimentological characteristics and depositional environments of two of these rock units: the SU and the HPU (Fig. 1C–E). To conduct this investigation we have used images from High Resolution Imaging Science Experiment (HiRISE), from the Mast-mounted cameras (Mastcams), and from Mars Hand Lens Imager (MAHLI) camera mounted on the arm of the Curiosity rover. Our study presents a documentation of large floods in Gale crater based on in-situ, rover-based observations.

## Results

The HPU and the SU are exposed in an 8 km by 8 km area on the northern areas of Aeolis Palus (Fig. 1C). They occur in well-defined stratigraphic order: The SU consistently overlies the HPU. In addition, both are overlain by the RTU throughout their exposures (Fig. 1C).

**Figure 2:**
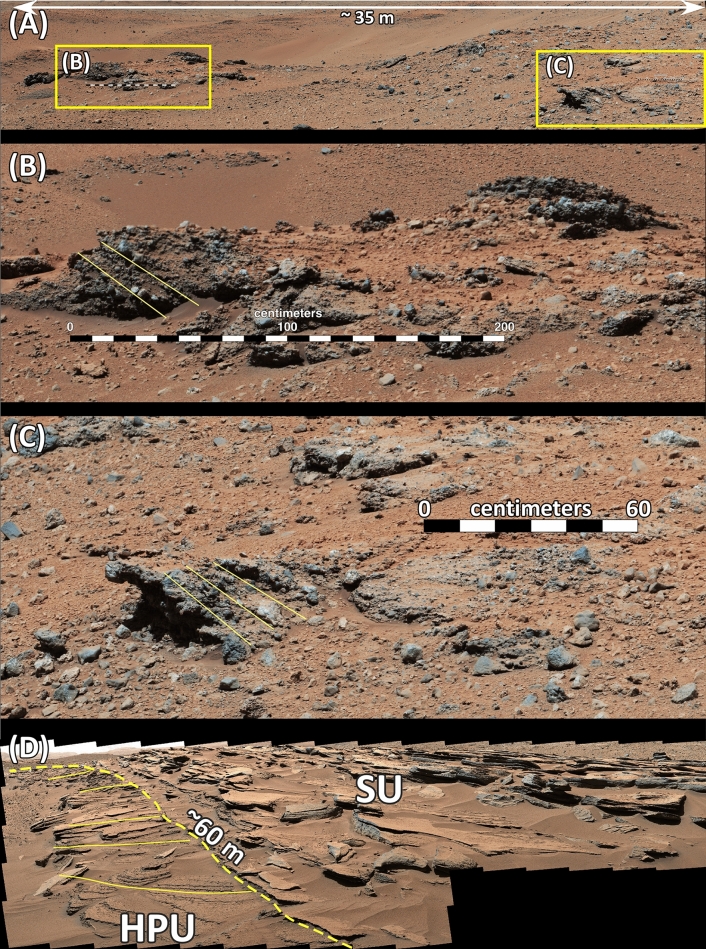
(**A**) Mastcam image mosaic (Mcam02747) acquired on Sol 646 (looking southeast) shows the exposure of the HPU inside an impact crater. Here the unit is about 8 m thick and consists of cross bedded unsorted conglomerate with clasts as large as 20 cm across. Cross beds indicate flow toward north (to the right of the image). (**B**) and (**C**) are enlarged portions of areas in (**A**) and show cross-bedded conglomerate. Yellow lines are trace of cross beds. (**D**) Mastcam image mosaic (Mcam02608) acquired on Sol 631 (looking northeast). The location of the image is shown in Fig. 1D. The image shows a 60 m-long cross bed in the HPU that is 2–3 m high. The Cross bedded layer is truncated by layers of the SU with a sharp contact shown by yellow dashed line. It indicates flow direction to northeast. Images used to generate mosaics of this figure are publically available at the Planetary Data System web site at https://pds-imaging.jpl.nasa.gov/ (see supplemental documents). Credit: Malin Space Science Systems and NASA/JPL.

**Figure 3:**
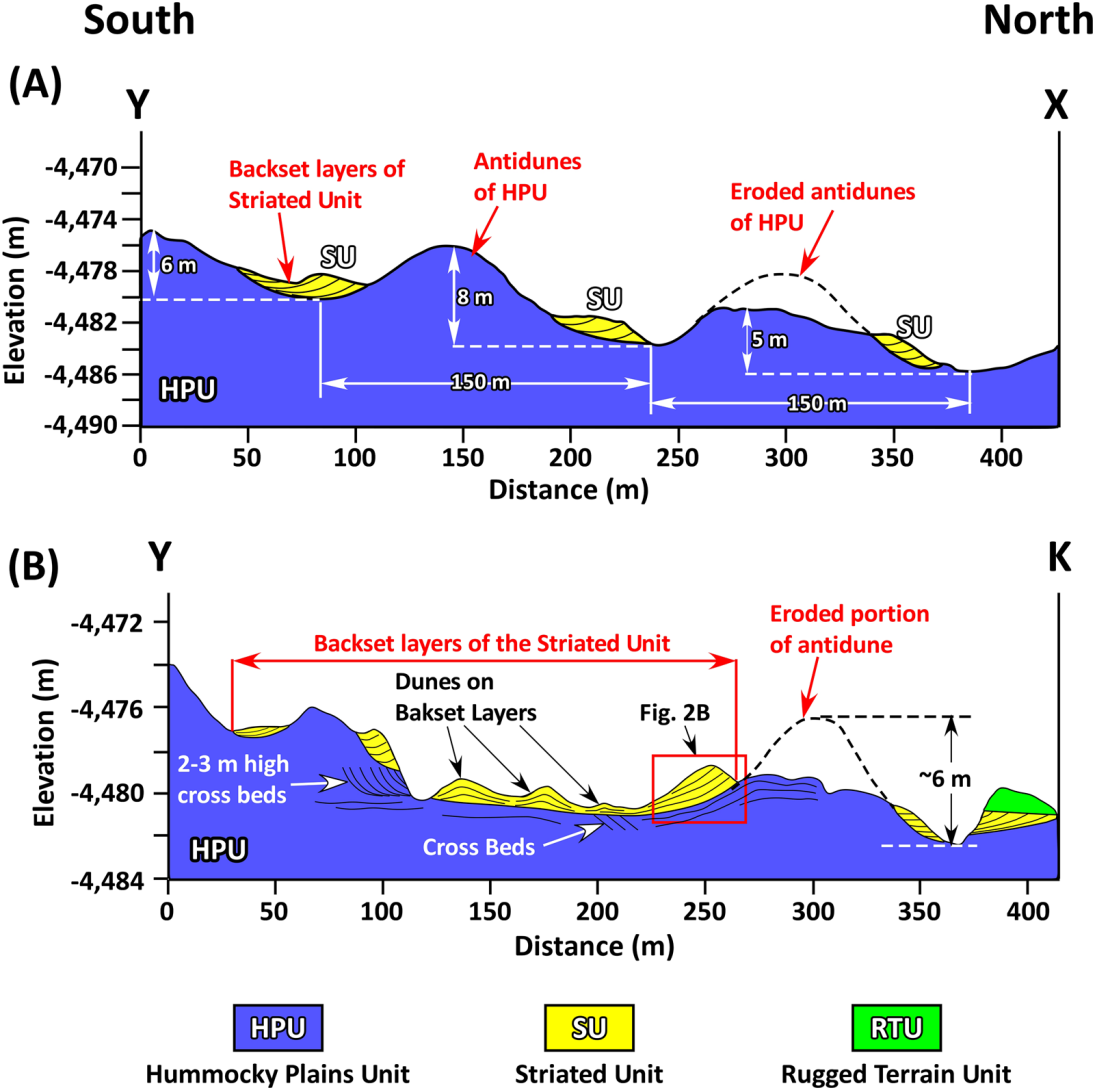
(**A**) North–south geological cross sections along lines X–Y (see Fig. 1C,D for the location). Ridges in the HPU are symmetrical and up to 8 m high along this line. Wavelength of ridges is 150 m. The SU exclusively occurs on the south-sloping flanks of these ridges and extends to troughs between them. (**B**) North – south geological cross section along K–Y (see Fig. 1D,E for the location). The cross section is along the rover’s traverse in the Kimberley region. It indicates that the ridge on which the SU deposited is moderately to highly eroded.

The HPU is distinguished by its dark gray tone, smooth surface, and sparse impact craters on HiRISE images (Fig. 1D). Lithologically, the HPU is composed of poorly sorted conglomerate with particles ranging in size from silt to boulders throughout its exposures along the rover’s path (Figs. 2A, S2). In few localities where its internal structure is exposed, the HPU displays large cross beds that are 2—6 m high and up to 60 m long (Figs. 2, S2, S3). The flow direction is directed toward north as inferred from the cross beds (Figs. 2A, S2). Most importantly, the HPU displays linear ridges which occur in variable states of preservations (Fig. 1D). These ridges are relatively straight, parallel, and about 500–800 m long in their exposed outcrops (Fig. 1D). Their crests trend at N60° E (Fig. 1D), are nearly flat, and appear to be eroded (Fig. 3). Ridges are symmetrical with each limb sloping about 4° to 9° (Fig. 3). The distance between these ridges is about 150 m (Figs. 1D, 3). Their present amplitude ranges from 5 to 10 m (Fig. 3). The base of the HPU is not exposed but the presence of 10 m-high ridges suggest it is a least 10 m thick (Fig. 3). These ridges are best preserved in the Kimberley region (Fig. 1D). They gradually disappear northward (Fig. 3).

**Figure 4:**
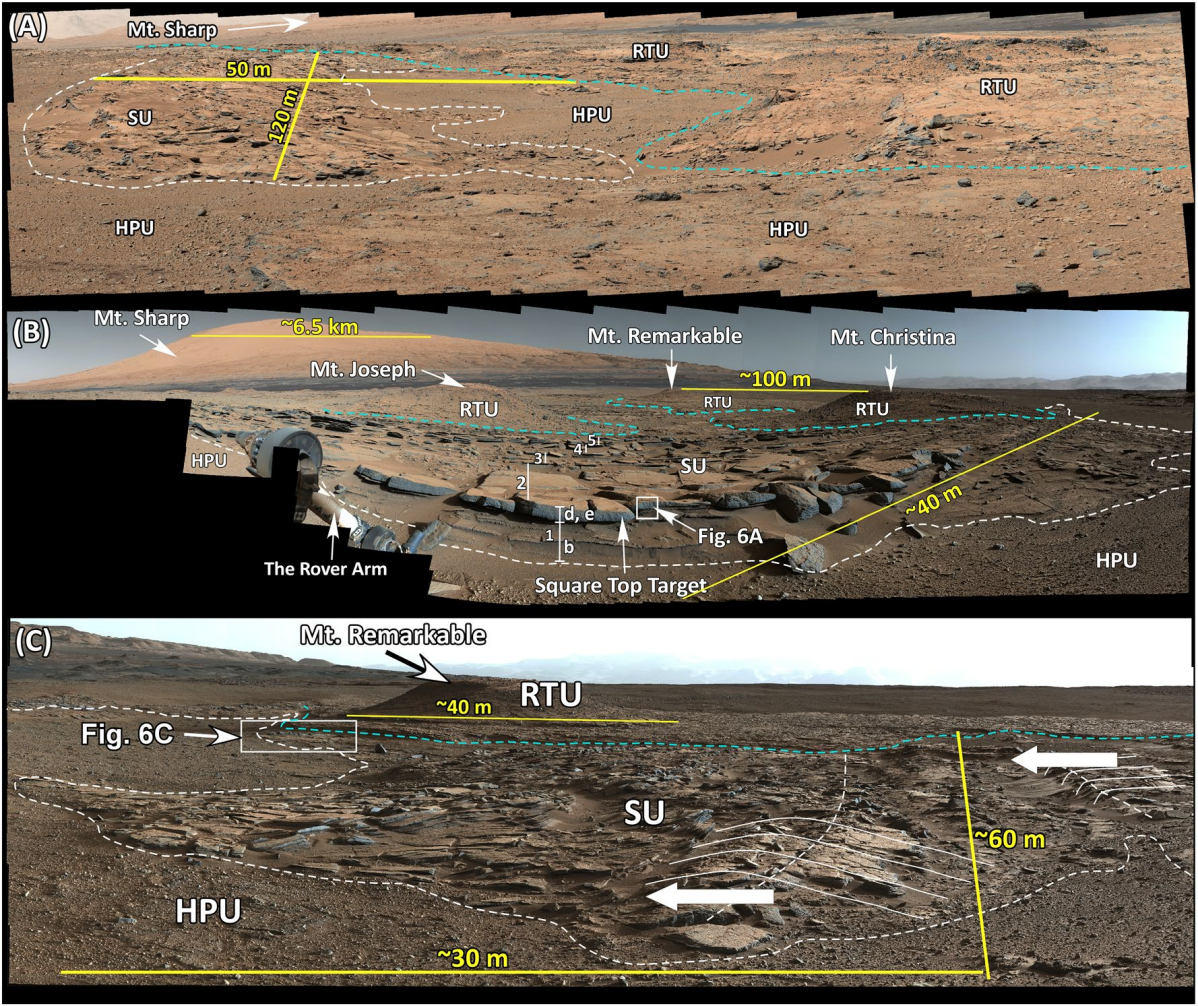
(**A**) Mastcam image mosaic (Mcam02230) acquired on Sol 551 (looking east) shows one of the patches of the SU and its south-dipping layers. This patch is 120 m along the strike direction and 50 m along the dip direction. The SU overlies the HPU with a sharp contact shown in white dashed line. The RTU overlies the SU and the HPU shown by the dashed cyan line. (**B**) Mastcam image mosaic (Mcam02407) acquired on Sol 580 (see Fig. 1D for location) shows another patch of the SU. This patch is 120 m along the strike direction and 100 m along the dip direction. The SU layers dip southward. The dashed white line shows the contact between the SU and the HPU. The dashed cyan line shows the base of the RTU and its contact with the SU and the HPU. (**C**) Mastcam image (mcam02484) acquired on Sol 590 shows the relationships between the HPU, the SU, and the RTU at the Kimberley region (see Fig. 1D for location). The view is toward the west. The SU overlies the HPU with a sharp contact shown with a dashed white line. The RTU overlies the SU and the HPU also with a sharp contact shown in dashed cyan line. Thin solid white lines mark secondary dunes formed in the SU. These dunes are between 30 and 150 cm high. White arrows show the inferred dune migration developed on layers of the SU. Images used to generate these mosaics are publically available at the Planetary Data System web site at https://pds-imaging.jpl.nasa.gov/ (see supplemental documents). Credit: Malin Space Science Systems and NASA/JPL.

The SU occurs in patches rather than as continuous layers (Fig. 1C). In contrast to other strata in Gale crater which are horizontal to nearly horizontal, patches of the SU consists of dipping layers that strike N60° E and have an estimated dip of about 10° SE (Figs. 4A,B, S4, S5). However, the dipping layers become conformable to the surface on which they deposited at about 50–100 m in a down dip direction (Figs. 4C, S6). Each patch is concave upward and extends 100–200 m along the strike and 50–100 m along the dip (Figs. 4A,B, S4, S5). The thickness of the SU in each patch is about 5–10 m.

Most importantly, the SU layers deposited on the south limbs of ridges of the HPU and extend to troughs between them (Fig. 3A). The strike of the SU is parallel to the trend of crests of symmetrical ridges, both at N60° E (Fig. 1D). The SU layers consistently dip at about 10ºSE southeast (Figs. 4A,B, S4, S5) which is similar to the measured 4°–9° southerly slope of limbs of the ridges on which it was deposited (Fig. 3A).

**Figure 5:**
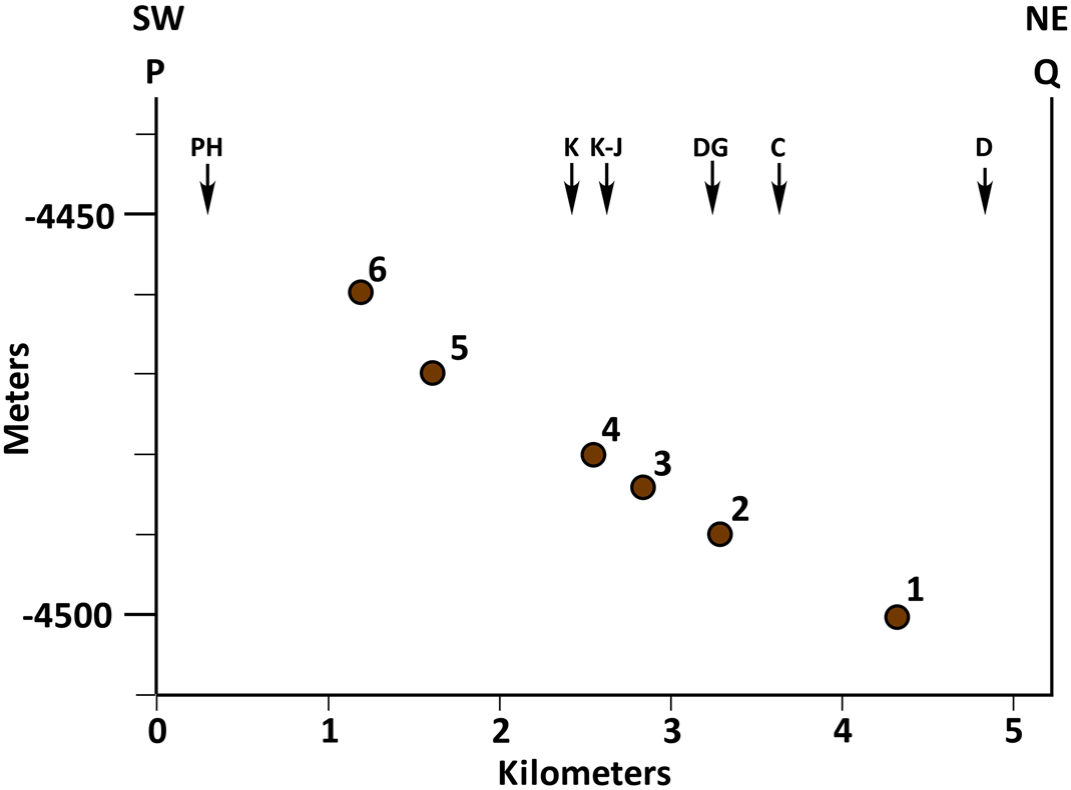
Cross section shows the elevation of the surface on which the SU deposited rises about 40 m along the 3 km exposure of this rock unit. Points used to draw the cross section as well as the P–Q line of cross section are shown in Fig. 1C. *C* Cooperstown, *D* Darwin, *DG* Dingo Gap, *J–K* Junda—Kylie, *K* Kimberley, *PH* Pahrump Hills.

The elevation of the surface on which the SU deposited is about − 4500 m near its northernmost exposures and − 4460 m at its southernmost exposure (Fig. 1C). This represents a rise of 40 m over 3 km of horizontal distance, or a slope of about 1º northward (Fig. 5). That is, the surface on which each patch of the SU deposited rises in elevation from north to south or from the landing site toward Mt. Sharp (Fig. .1B,C). This is also clearly demonstrated by the cross section at the Kimberley area (Fig. 1D). Here, the base of the SU patches rises 6 m in elevation over 300 m of horizontal distance, also indicating that the surface on which the SU patches deposited had a slope 1.2º northward (Fig. 3A).

**Figure 6:**
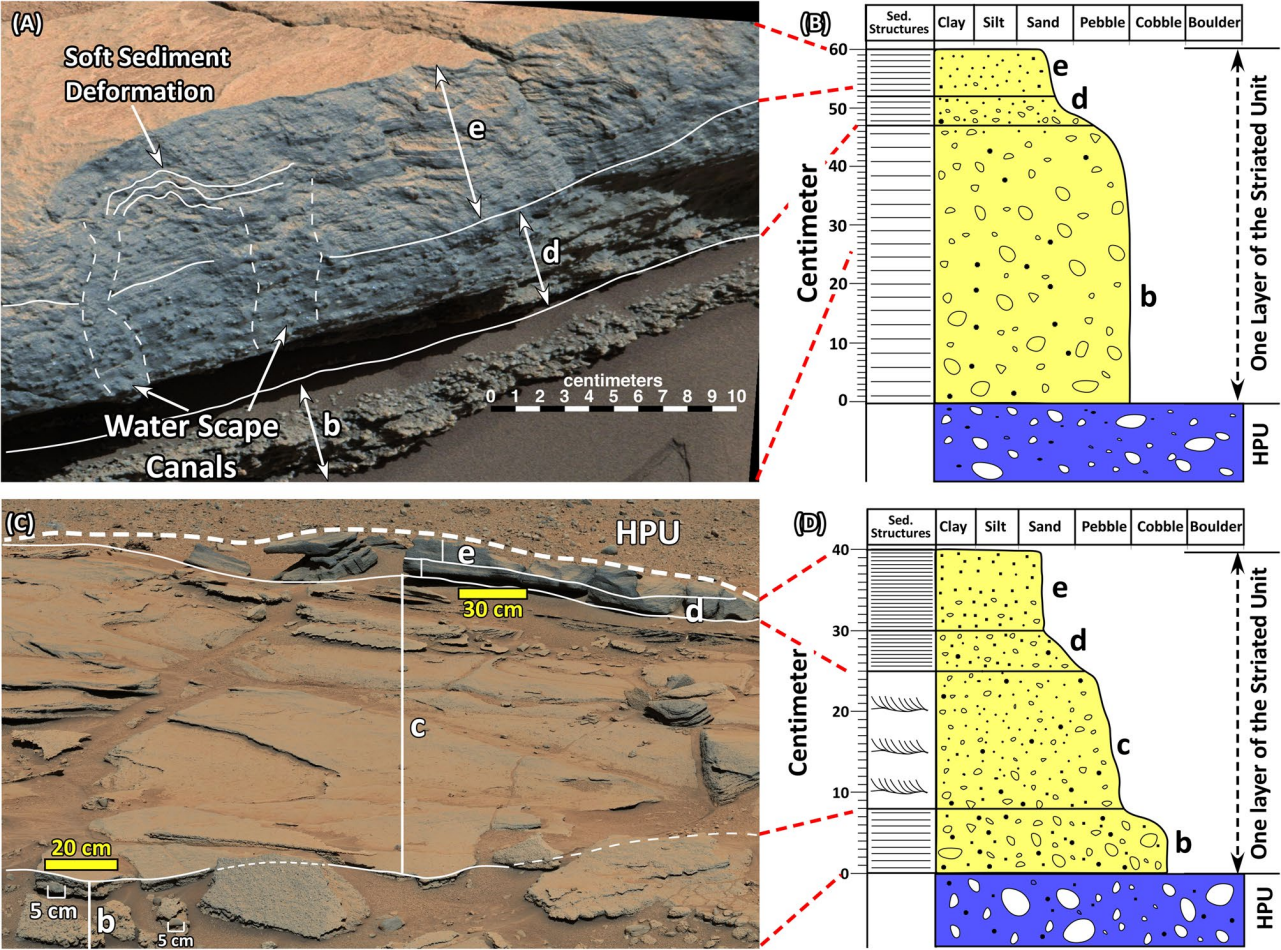
(**A**) Mastcam image mosaic (Mcam02446) of the Square Top target acquired on Sol 586. It shows the lithology of the top portion of layer 1 in Fig. 4B. Only the uppermost part of the thin-bedded conglomerate (the b interval) is shown in this image. It grades upward to the poorly sorted pebbly sandstone (the d interval) with a thin transitional interval. The d interval grades upward into the laminated sandstone (the e interval). Evidence of soft sediment deformation and possible water escape structure are shown in the e interval. (**B**) The stratigraphic column of the layer 1 shown in Fig. 4B. This layer overlies the conglomerates of the HPU with a sharp contact shown in Fig. 4B. The basal part of the image consists of thin-bedded conglomerate (the b interval). It is overlain by a graded-bed interval consisting of pebbly sandstone (the d interval) that transitions upward to a laminated sandstone (the e interval). (**C**) Mastcam image mosaic (Mcam 02540) of another layer of the SU at the Kimberly region acquired on sol 603 (See Fig. 4C for location). This image was taken looking south. (**D**) The stratigraphic column on the layer shown in (**C**). This layer also overlies conglomerate lithology of the HPU with a sharp contact, visible in Fig. 4C. This layer begins with 8 cm of conglomerate (the b interval) that is overlain by 17 cm of cross-bedded conglomerate (the c interval) which is in turn overlain by 5 cm of bedded pebbly sandstone (the d interval). The topmost part of the layer consists of 10 cm of laminated sandstone (the e interval). Images used to generate these mosaics in (**A**) and (**C**) are publically available at the Planetary Data System web site at https://pds-imaging.jpl.nasa.gov/ (see supplemental documents). Credit: Malin Space Science Systems and NASA/JPL.

Two layers of the SU were examined closely by the rover at two different locations. In one area investigated on Sol 586, the SU layer begins with about 60 cm of conglomerate (the b interval). It overlies the conglomerates of the HPU with a sharp but conformable contact (Figs. 4B, 6A,B, S5, S7). The conglomerate of the b interval is thin bedded, grain-supported, and consists of grains ranging in size from 2 to 5 mm (Figs. 6A, S7).

The b interval is overlain by a distinct, 13 cm-thick, normally graded bed with a sharp decrease in grain size (Figs. 6A, S7). The base of the graded bed consists of 5 cm of pebbly sandstone (the d interval) that is grain supported and poorly sorted (Figs. 6A,B, S7).

**Figure 7:**
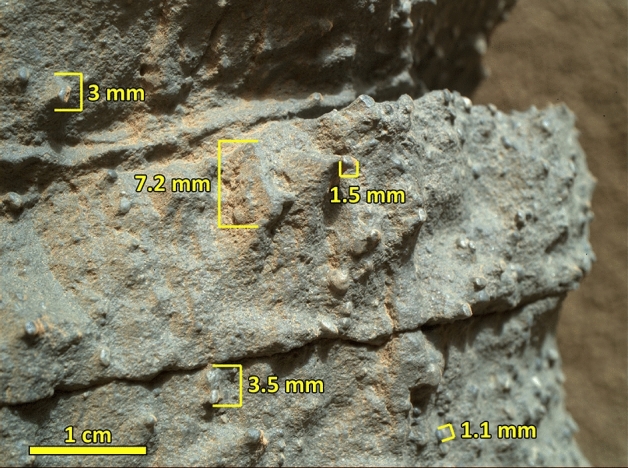
A close-up image taken by Mars Hand Lens Imager (MAHLI) camera (0585MH0002970010202888C00) shows characteristics of the graded bed interval in the Square Top target acquired on Sol 585. Highly rounded coarse sand and pebble fragments float in medium grain sand. Credit: NASA/JPL-Caltech and Malin Space Science Systems.

The d interval grades upward to 7–8 cm of laminated, medium grain sandstone (the e interval) that shows irregular laminations (1 mm to 2 mm thick), and displays soft sediment deformation (Figs. 6A, S7). Laminations are disrupted at two locations on the left side of the image (Figs. 6A, S7). A MAHLI image of the graded bedded interval shows that it is highly unsorted but consists of grains that are well-rounded and dark in color (Fig. 7). Five layers of the SU, each terminating with the distinct graded bed capping unit, are visible on the Mastcam image at this locality (Figs. 4B, S5).

Another layer of the SU was studied on Sol 603 (Figs. 6C,D, S8). Here too, the SU directly overlies the conglomerate of the HPU with a sharp contact without any transitional layers (Figs. 4C, S6). The basal 8 cm of this layer of the SU consists of grain supported, unsorted, thin-bedded conglomerate: the b interval. Some of its fragments are 5 cm long (Figs. 6C, S8). The b interval grades upward to about 17 cm of cross-bedded conglomerate with grains ranging in size from 2 to 4 mm: the c interval (Figs. 6C,D, S8). A distinct 15 cm-thick normally graded interval also caps this layer (Figs. 6C,D, S8). Its characteristics cannot be resolved as clearly as the one seen on Sol 585 (Figs. 6A,B, S7). But the basal 5 cm consists of pebbly sandstone, the d interval, followed by 10 cm of laminated medium grain sandstone: the e interval (Figs. 6C,D, S8).

## Discussion

The previous sedimentological study suggested a fluvial-deltaic-lacustrine depositional system for strata examined by the Curiosity rover between the landing site and the Pahrump Hills location^12^. The model advocated that melting of ice and snow on the northern rim of Gale crater generated liquid water that flowed southward and drained into a shallow lake when Gale crater was nearly empty^12^. The proposed system resulted in deposition of four lithologies each representing a specific environment: lacustrine, clinoform (foreset beds) of a delta, fluvial, and alluvial fan (Fig. S9).

The model clearly equates the mudstones of the MF to deposition in a lake or a lacustrine environment (Fig. S9). It also presents the SU as the clinoform (foreset beds) of a delta. Authors of this model^12^ do not use terms such as the RTU or the HPU in their proposed depositional environments. However, they describe sedimentological characteristics of sandstone outcrops mapped as the RTU and interpret them as river deposits^12^. Similarly, they describe conglomerates mapped as the HPU and interpret them as deposition in an alluvial fan environment^12^. Therefore, the four lithologies of the study area namely the MF, the SU, the RTU, and the HPU are equated to four depositional environments such as the lucustrine, delta, fluvial, and alluvial, respectively^12^ (Fig. S9).

The proposers of the fluvial-deltaic-lacustrine model considered these four rock units to be time-equivalent^12^; that is, their deposition evolved side-by-side through time. After the lake dried up, these four rock units were buried by about 5 km of sediment that filled Gale crater. They were then exposed along an erosion surface (Fig. S9) when margins of Gale crater were excavated creating the modern morphology of the Gale crater^12^. According to this model, deposition of all four rock units predated the formation of Mt. Sharp^12^.

We have found four major discrepancies between our observations and the proposed fluvial-deltaic-lacustrine depositional environment that was applied to Gale crater^12^. First, this depositional system is very common on Earth^35^, as was also pointed out by the proposers of the model^12^. As a result, we have a clear understanding of its sedimentary record^35^. Basin-ward migration of time equivalent environments of a fluvial-deltaic-lacustrine system on Earth produces a coarsening upward stacking pattern^35^. It consists of lacustrine mudstone at the base which transitions upward to deltaic strata, then to fluvial deposits, and is finally caps by alluvial layers^35^. This stacking pattern is shown schematically to exist in Gale crater (Fig. S9), but it has not been seen anywhere along the rover’s path.

Second, the key component of the fluvial-deltaic-lacustrine interpretation is the SU: the rock unit that was interpreted as a clinoform component (foreset beds) of a delta and is the main focus of our investigation. According to the model, the deltaic clinoform (i.e. the SU) transitioned into the lacustrine mudstone of the MF (Fig. S9). Therefore, the SU layers should overlie the lacustrine mudstones of the MF as the system migrated basinward (Fig. S9). But the SU neither transitions nor overlies the MF anywhere along its exposures imaged by the rover (Fig. 1C). In fact, the SU has never been seen to transition into any rock unit. It consistently overlies the conglomerates of the HPU and underlies the sandstones of the RTU both with sharp contacts (Figs. 3, 4).

Third, patches of the SU occur at successively higher elevations from north to south, that is, from the landing site to Mt. Sharp (Figs. 1C, 5). This characteristic was also noted by previous investigators^12^. They suggested that each exposure of the SU is a small delta that deposited successively at high elevations due to the continued rises in lake level^12^. That is each patch of the SU becomes younger southward (Fig. S9). We demonstrated that the occurrence of the SU at higher elevation southward was because it deposited on a surface that was rising in elevation in that direction (Fig. 5). It was not because the SU became younger southward due to rises in lake level. Such an interpretation would have required the SU and the MF to deposit up-section together as shown schematically to have occurred by these authors (Fig. S9). But the SU has never been seen in contact with the MF along its exposures. In fact, dipping layers of the SU at each patch become tangential to the surface on which they deposited (Figs. 4C, S6); but they neither overlie nor transition into lacustrine mudstone of the MF (Fig. 4C, S6). In fact, the SU does not transition into any other unit along its exposures. As such, each occurrence of the SU could not have been a separate delta that was deposited at higher elevation during rises in lake level.

Lastly, the giant cross beds of the HPU (Fig. 2) suggest that the flow that deposited this rock unit was towards north. Combined with the north-sloping surface on which the SU was deposited, these indicate that the SU and the HPU formed on a north sloping surface. The only north sloping surface in this area is the northern flank of Mt. Sharp. That means that both the HPU and the SU were deposited after the emergence of Mt. Sharp. Another investigation has already documented that the RTU (the rock unit that overlies both the HPU and the SU) was also deposited after the emergence of Mt. Sharp^9^. This means that three out of four components of the fluvial-deltaic-lacustrine interpretation (the SU, the HPU, and the RTU) formed after the emergence of Mt. Sharp, not before it as previous investigators suggested^12^. Therefore, the reason the SU has not been seen in contact with the MF anywhere along its exposures is because the two rock units formed in two different times. One (the MF) was deposited when the crater was empty, then buried by 5 km of strata, and was subsequently exposed when margins of Gale crater were excavated resulting in the emergence of Mt. Sharp. The other three (the SU, the RTU, and the HPU) formed after the emergence of Mt. Sharp when Gale crater had acquired its modern morphology.

These major differences between our observations and the existing interpretation have persuaded us to re-examine the sedimentation history of strata examined by the Curiosity rover in Gale crater in general and the depositional environments of the individual rock units in particular. This contribution primarily addresses the depositional environments of the SU and, secondarily, that of the HPU, as it relates to the main goal of this study.

### Proposed Interpretation

Our alternative interpretation for the depositional environments of the SU is based on three characteristics of this unit. They are: (1) the sedimentological features of its layer, (2) the geometry of its occurrences and the southerly dips of its strata, and (3) its relationship with the rock unit that underlies it.

Sedimentologically, the two layers of the SU that were investigated by the rover show systematic upward changes in lithology, grain size, and sedimentary structures (Fig. 6). Each layer begins with a thin-bedded conglomerate (the b interval) consisting of granule to coarse pebble size fragments (Fig. 6). Thin layering and the grain-supported texture suggest deposition as bedload. Their sharp plane bedding indicates that they were deposited under a condition commonly referred to as the upper flow regime^36^.

The b interval is overlain by a cross bedded granule conglomerate, the c interval (Fig. 6C,D). The decrease in grain size and the change in sedimentary structures from sharp plane layering to cross bedding indicate a decrease in flow strength or a transition from upper to lower flow regime conditions^36^.

The cross bedded c or the thin-bedded b intervals are followed upward by a normally graded capping layer (the d and the e intervals) across a sharp transitional boundary (Figs. 6A, S7). A rapid reduction in grain size, co-deposition of pebble and sand grains, the absence of cross beds, and irregular laminations indicate that the transition from the c (or the b) interval to the d interval involved a rapid and sudden decrease in flow strength^36^. It resulted in co-occurrence of well-rounded granule, small pebble, and sand-size grains (Fig. 7), suggesting that the flow was so weak that deposition was most likely occurred from suspension. Upward grading of pebbly sandstone (the d interval) to a sandstone interval (the e interval) with irregular laminations suggests suspension sedimentation continued until the end of the deposition of the normally graded bed (Fig. 6). The presence of soft sediment deformation and water escape structures (Figs. 6A, S7) indicate deposition under subaqueous conditions.

Morphologically, the SU occurs as patches rather than as continuous layers (Figs. 1C, 3). In contrast to other strata in Gale crater which are horizontal, layers within patches of the SU have a strike of about N60° E and a dip of about 10° SE (Figs. 4A,B, S4, S5). However, the dipping layers become tangential to the surface on which they were deposited within about 50–100 m in a down-dip direction (Figs. 4C, S6). These patches are 5–10 m thick, concave upward, and are 100–200 m in the strike direction and 5–100 m in the dip direction (Figs. 4A,B, S4, S5).

Patches of the SU occur exclusively between ridges of the HPU conglomerate (Figs. 1D, 3). Most importantly, the strike of the SU layers and the trend of the ridge crests in the HPU are both at about N60° E (Fig. 1D). In addition, the dip of the SU strata is similar to the slope of the limb of the HPU ridge (4°–9°) on which they were deposited (Fig. 3). These relationships strongly suggest that the deposition of the SU is related to the formation of ridges of the HPU. Furthermore, at least in one location along the rover’s path in the Kimberley region, the SU sharply truncates and overlies a 60 m-long, 2–3 m thick giant cross bed within the HPU (Figs. 2D, S3).

Systematic changes in lithology, the upward decrease in grain size, and the sequence of sedimentary structures suggest that each layer of the SU is a single sedimentation unit that was deposited by a waning flow. Deposition began with a flow that was strong enough to transport grains as large as coarse pebbles as bedload (the interval b) and terminated by a rapid decrease in flow strength so that sand and granules deposited from suspension (intervals d and e). These characteristics are traditionally attributed to deposition as turbidites: a type of sediment gravity flow deposits in which sediments are kept in suspension by flow turbulence^37,38^. Turbidites are very common in deep water environments of modern and ancient oceans and lakes on Earth^37,38^.

In addition, layers with features similar to those found in the SU have also been documented by waning catastrophic glacial floods of the Pleistocene Epoch on Earth and commonly referred to as rhythmites^39–41^. This is particularly apparent when floods drain into lakes, best recorded in Lake Lewis that formed along the path of the Missoula Lake floods^40^.

The absence of strata indicative of deepwater lacustrine sedimentation within the SU layers challenges the turbidite and rhythmite interpretations. In addition, neither of the two hypotheses satisfies occurrences of the SU as concave upward patches of dipping layers. Furthermore, the turbidite and rhythmite hypotheses do not account for the deposition of the SU between ridges of the HPU (see Figs. 1D,E, 3A). Nevertheless, it is clear that layers of the SU were deposited by waning flows; but they are neither true turbidites nor true rhythmites.

Three lines of evidence suggest that the deposition of the SU patches was related to the formation of 10 m-high ridges of the HPU. First, the SU occurs exclusively between these ridges and specifically on their south limbs. This is best expressed in areas where these ridges are well preserved such as the Kimberley region (Figs. 1D,E, 3A). Second, the strike of the SU layers is identical to the trend of ridge crests of the HPU both at N60°E (Figs. 1D,E); and third, the value and the direction of the dip of the SU layers mimics those of the slope of the southern limb of the ridge on which they deposited (Fig. 3A).

These relationships suggest that the deposition of the SU is directly related to the origin of the ridges of the HPU. These ridges are parallel and symmetrical (Figs. 1D, 3A). They have a consistent wavelength of about 150 m and amplitudes that range from 5 to 10 m (Figs. 1D, 3). They consist of unsorted conglomerate with fragments as large as 20 cm across (Fig. 2). In few locations where their internal structures were preserved, they display 2–5 m high, 60 m long cross beds, particularly in their basal part (Figs. 2D, S3). The topmost part to the ridge consists of thin-bedded conglomerate, however. Formation of these ridges cannot be attributed to wind erosion after the deposition of the HPU. This is because wind could not have removed cobbles and boulders of the HPU to carve perfectly symmetrical ridges at identical spacing and at the same time leave behind sandstones of the SU layers. In contrast, lithology, morphology, giant cross beds, and thin bedding suggest that ridges of the HPU are giant bedforms produced by moving water. Bedforms of this size and their lithology are characteristics of deposition by huge floods similar to those formed by Pleistocene floods on Earth^42–44^. They are referred to as giant current ripples, but are actually giant gravel dunes^42–44^. However, giant bedforms (ridges) of the HPU are symmetrical (Figs. 1D, 3); and bedforms with these characteristics are antidunes not dunes^36,45–48^. They are also known as stationary waves or standing waves^36,45–48^.

**Figure 8:**
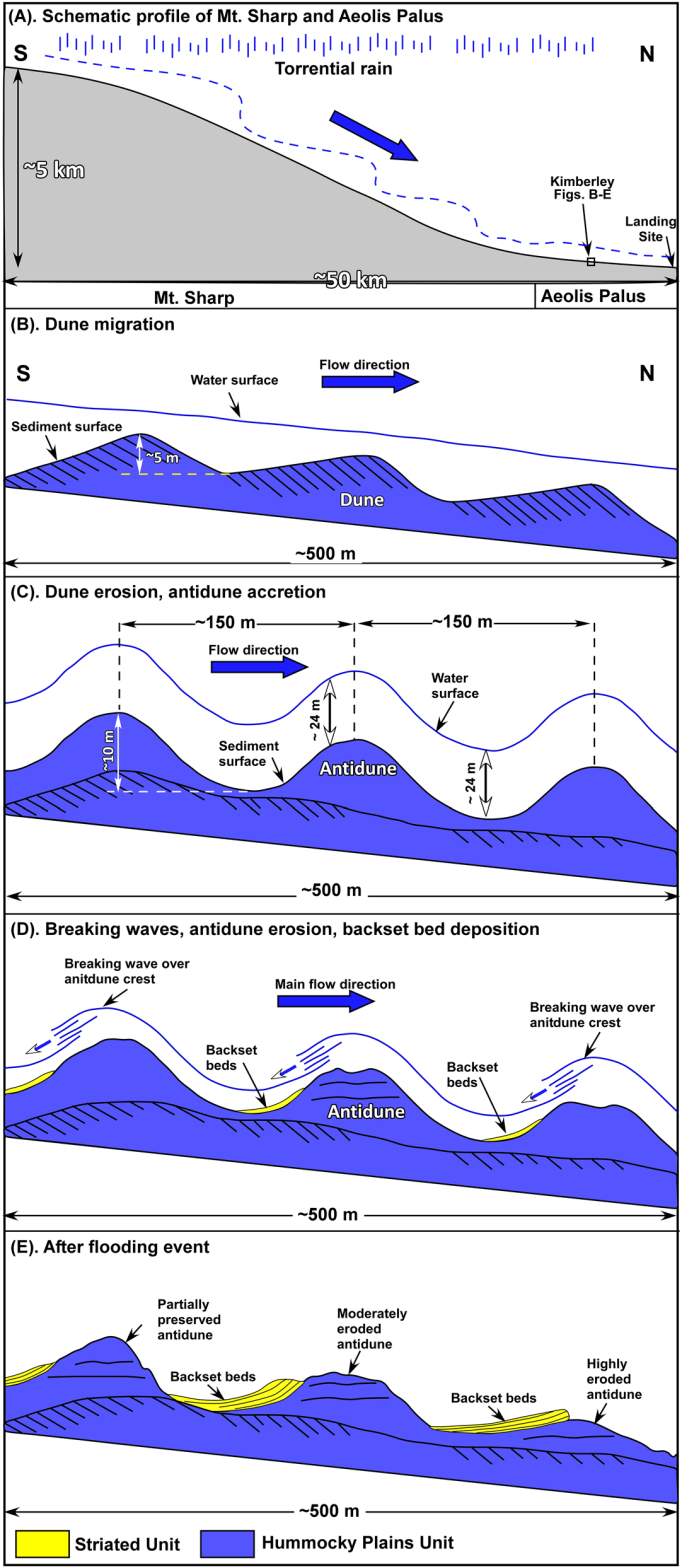
Schematic diagram shows the location of the study area as well as the evolution of antidunes of the HPU and its relations to the deposition of the SU. (**A**) Schematic diagram show the location of antidunes relative physiographic features of Gale crater. Heavy rainfall flowed downhill from Mt. Sharp and combined with waters coming from the Southern Highlands to form flash floods that deposited the HPU and the SU on Aeolis Palus. (**B**) The presence of 2–5 m high cross beds in deeply eroded ridges suggest that the antidune ridges of the HPU started as giant gravel dune that migrated downhill toward north as did flood waters. (**C**) As flooding continued, gravel dunes were partially to completely eroded and were replaced by 10 m-high symmetrical antidunes. (**D**) When flooding waned, water broke over antidunes, eroding sediments from their crests and re-deposited them on the up-flow limb of antidunes as south-dipping patches of fining upward layers. (**E**) Stratal relationship after flooding ended. Waning must have been rapid because antidune were partially preserved in up-flow areas, but highly to completely eroded in down-flow regions.

Antidunes form when flow is strong enough to remove previously deposited sedimentary structures such as dunes and high energy plane laminations^36,45–48^. Sedimentation, erosion, and migration of dunes are very different from those of antidunes. In dunes, erosion occurs on the stoss side, deposition takes place on the lee side, and migration is in the same direction as the flow. In antidunes, erosion occurs on the lee sides, deposition on the stoss side. Antidunes migrate down-flow, up-flow, or remain stationary^36,45–48^. In addition, the sediment surface and water surface are out of phase in dunes but in phase in antidunes (Fig. 8C). When flow depth decreases, water breaks on antidune crests and partly to completely erodes them, depositing removed sediments on flanks and troughs of antidunes as backset beds^36,45–48^. As a result, antidunes are rarely preserved in the rock record except when flow wanes rapidly^36,45–48^. Flow that breaks over the crests of antidunes is sediment-saturated and loses its strength quickly, leading to the deposition of fining upward layers which are identical to those found in turbidites^37,38^ or rhythmites^39^^—^^41^. The main difference is that backset beds occur as dipping patches because of deposition on sloping flanks of anitdunes; whereas true turbidites and rhythmites occur as continuous, horizontal layers^37–41^. In essence, we have discovered a new environment and a new mechanism in Gale crater, Mars, where layers with sedimentological characteristics identical to turbidite deposited by processes other than sediment gravity flows.

Indeed, the 10 m-high antidunes of the HPU that consist of cobble- and boulder-size fragments indicate fast-moving flows such as floods. However, the presence of 2–5 m high cross beds in few locations suggests these floods initially deposited as giant gravel dunes (Figs. 2D, 8B). As flooding continued and flow gained additional strengths, gravel dunes were partly to completely eroded and were replaced by 10 m-high antidunes (Figs. 3A, 8C). The remnants of eroded giant dune cross beds were seen in several locations along the path of the rover (Fig. 2D). When flooding waned, water broke over the crests of antidunes ridges of the HPU and began to erode them (Fig. 8D). Eroded sediments were re-depositing on the up-flow limbs (southern flanks) of antidune ridges as south-dipping patches of backset beds (Figs. 3A, 8E). The erosion of antidune ridges was variable along the flow path which was from south to north. In the southern up-flow regions (high elevations), antidunes suffered minor erosion and were well preserved possibly because the rapid waning exposed these ridges preventing their erosion (Figs. 3A, 8C). In the northern down-flow areas (low elevations), however, ridges experienced major erosion to the point that they are not easily identifiable (Figs. 2B, 4A, 8E). They were totally eroded north of the Dingo Gap region (Fig. 1C).

The southerly dips of the backset beds gave the false impression that the main flow direction was from the north to the south delivering sediments from the northern rim of the crater to form a clinoform delta in a lake near the center of Gale crater^12^. However, this study demonstrated that southerly dips of the SU layers was due to their deposition on south-facing limb (up-flow flanks) of antidunes as backset beds when the main flow was northward delivering sediments from upland regions (Mt. Sharp), to lowland areas northward (Fig. 8A). Initially, backset flows were strong enough to carry coarse pebble fragments as bedload and leave behind secondary bedforms which were up to 1.5 m tall (Fig. 4C). However, the rapid transition to a graded bed layer suggests that backset flow velocity decreased quickly so that granule, small pebbles, and sands deposited from suspension in the capping graded beds of each layer (Figs. 6,7). This interpretation suggests that the SU and the UPU formed during the same event, and that event was a gigantic flash flood on the northern flank of Mt. Sharp (Fig. 8).

Backset beds of the size and magnitude seen in Gale crater, Mars, have not yet been discovered in Earth’s fluvial depositional system. However, centimeter-size backset beds identical to those of Gale crater have been reported in ancient rocks and modern rivers on Earth^49,50^, and have been extensively documented in flume studies using fine sand grains^45–48^.

Wave length (L) of antidune ridges in the HPU can be used to estimate flow depth (h) and mean flow velocity (U) via Eqs. (1) and (2), respectively^45–48^:

1 $${\text{L}} = 2\pi {\text{h}}$$

2 $${\text{U}}^{2} = {{{\text{Lg}}} \mathord{\left/ {\vphantom {{{\text{Lg}}} {2\pi }}} \right. \kern-\nulldelimiterspace} {2\pi }}$$

where g is the Martian acceleration due to gravity (3.72 m/s^2^)^51^. The wave length of antidunes in the Kimberley region is about 150 m suggesting that flow depth at this location was about 24 m and that flow velocity was about 9.5 m/s.

A 24 m deep flood at the Kimberley region would have extended for 11 km from the crater wall to the west and Mt. Sharp to the east. This is consistent with the distribution of the HPU in this area of Gale crater (see Fig. 1C). As such, the discharge of flood would have been about 2.75 10^6^ m^3^/s. We consider our calculate discharge as a minimum. However, its exact value will not affect our results because our conclusions for the presence of floods in Gale crater are based on physical sedimentology. The discharge would have depended on the amount of water vapor in the Martian atmosphere at time of flooding (see below). It is important to note that our calculated discharge for floods of Gale crater is 7–10 times lower than the discharge for the Pleistocene floods on Earth such as Missoula Lake and Altai Mountain floods^2^ calculated to be about 10^7^ m^3^/s, and 100–1000 times lower than discharge through major outflow channels on Mars^2^ estimated to range from 10^8^ to 10^9^ m^3^/s.

## Source of flood waters

Mars is presently so cold (average temperature of – 60 °C) that water freezes and ice subsequently sublimates^52^. In fact, low solar luminosity would have kept Mars below freezing temperatures during its entire history regardless of how much green houses gases such CO_2_ were present^53–56^. Despite the cold climatic condition, abundant geological, morphological, and sedimentological features indicate that liquid water flowed on Mars and in some cases accumulated in lakes^1,12,34,57,58^. Therefore, the question of how liquid water formed has been debated extensively^59,60^. This means that we either need a mechanism to warm Mars to above freezing temperature or prevent water from freezing under a cold planetary condition. Suggestions include geothermal heating^1,61^, CO_2_ clouds^62^, asteroid impact^63^, high water salinity^64^, volcanism^65^, orbital changes^66,67^, and high methane concentration^68–71^.

At the present state of knowledge all of these mechanisms are plausible. Additional in-situ observations and data are needed for a definitive solution to how liquid water formed and flowed on a cold planet like Mars. Nevertheless, two processes appear to be more likely than others to have produced the giant floods that caused deposition of the SU and the HPU in Gale crater. The first is the conceptual model proposed to explain floods through outflow channels of Mars^1^. According to this scenario, the cold and dry Mars was punctuated by intervals of geothermal heating associated with volcanic activities. The event melted glaciers, similar to the generation of Icelandic floods^72^, and resulted in a rapid release of liquid water which flowed to Gale crater via a series of ancient drainages on the Southern Highlands. Water vapor combined with abundant CO_2_ and CH_4_ released from their solid reservoirs produced a warm and wet climate^1^.

The second possibility is the warming of Mars by a giant impact that vaporized glacial ice, melted solid CO_2_ reservoirs, and released a large amount CH_4_ from gas hydrate sources^63^. Subsequent cooling would have resulted in condensation of water vapors to produce planet-wide torrential rain^63^.

The warming by an impact is a more likely to have triggered floods of Gale crater than heating by a geothermal event. This is because over seven years of investigation, no evidence of ice-covered lakes such as dropstones or glacial deposits have been found in lacustrine strata of Gale crater^8–20^. This suggests that not only the source region was warm enough to generate liquid water but the sink area where water accumulated was also warm preventing water from freezing. This requires an instantaneous planet-wide heating event that would have resulted from a large impact^63^.

A giant impact may have punctuated the cold and dry climate of Mars and created an interval of a warm and wet one. A large volume of water vapor was generated from frozen reservoirs. Subsequent cooling resulted in a period of planetwide torrential rain. Runoff would have flown downhill from vast areas of the Southern Highlands and poured into a bowl-shaped, tilted depression such as Gale crater basically from all sides. This water would have combined with that flowed downhill from Mt. Sharp producing gigantic flash floods that deposited the SU and the HPU on Aeolis Palus (Fig. 8).

As such, the flood discharge was not limited by the catchment area in Gale crater; rather, it would have been controlled by the volume of water vapor in the atmosphere which itself depended on the total amount of water available on Mars at that time. Estimated water budget of Mar ranges from 34 to 550 GELs (global equivalent layer) of water^73^. We consider a value of about 150 GELs of water, estimated by hydrogen isotope analysis^74,75^, as a reasonable minimum approximation. This indicates that the Late Noachian had at least 5 times more water than the present-day Mars, which is estimated to be 34 GELs^52,76^. If all of the 150 GELs of water was put into the atmosphere as water vapor, then gigantic flash floods of Gale crater were the most likely outcome. In fact, sufficiently large liquid water would have been available to accumulate on Northern Lowlands forming Oceanus Borealis^77–79^.

The flooding event may have lasted only a few days. But, the warm and wet period persisted for much longer. This is because flood deposits of Gale crater (the SU and the HPU) are overlain by the RTU throughout their exposures (Fig. 1C). The basal strata of the RTU consist of thin-bedded fine grain sandstone^9^ and have been interpreted as a lacustrine sedimentation^80^. This suggests that the warm and wet conditions continued even after the flooding ended. However, its duration cannot be determined by our study. It may have lasted from few weeks to few centuries depending on the size of the impactor and the amount CO_2_ and CH_4_ in the atmosphere^63^; but it was certainly not long enough to cause the complete removal of unstable minerals by chemical weathering, leading to their preservation in Gale crater strata^81–83^. It will take longer than a few decades or several centuries of warm and wet condition to remove all unstable minerals^84^.

Since this warming event occurred when Gale crater had acquired its modern morphology, torrential rain may have also carved several huge canyons on Mt. Sharp (Fig. 1A). One of them, called the Grand Canyon of Gale crater, is 2 km wide and 200–300 m deep (Fig. 1A). It is also very likely that flooding of Gale crater was a part of the proposed intense fluvial activity that affected the entire Mars at the end of the Noachian period^32^.

## The age of the flooding and the potential impact that triggered it

Neither the SU nor the HPU have any features that can be used to determine the timing of their deposition. But, both rock units consistently underlie the RTU throughout their exposures (Fig. 1C). The top surface of the RTU is so heavily cratered that it was initially mapped as the cratered surface^11^. This indicates that deposition of the RTU occurred when the heavy bombardment of Mars had not ended. This event is attributed to the boundary between the Noachian and the Hesperian periods of the Martian history and considered to have occurred around 3.6 billion years ago^85^. Therefore, this relative dating indicates that flooding had already occurred prior to the end of Noachian Period. That is, floods are older than 3.6 billion years.

Our relative dating of floods is supported by a crater count study^33^. The HPU, the SU, and the RTU occur on the northern part of the crater floor or on Aeolis Palus (Fig. 1C). With all likelihood, they are the same strata that were named the crater floor layer whose top surface yielded a crater count date of 3.6 billion years^33^.

Therefore, floods occurred after the formation Gale crater but before 3.6 billion years ago. Unfortunately, there is a considerable uncertainty about the age of impact craters on Mars depending on methods used^34^. For example, the proposed age for Gale crater ranges from the Early Noachian to Early Hesperian, a difference of over 400 million years^34^. A recent study suggested that the most likely age of Gale crater is the Middle Noachian^34^. On the basis of these constraints, we tentatively suggest that flooding is not younger than 3.6 billion years and not older than 3.85 billion years. Therefore, we searched for impact craters within this age range. In addition, the triggering impactor must have been large enough to initiate a planet wide warming to melt frozen reservoirs of H_2_O, CO_2_, and CH_4_. As such, we limited our search to impactors which produced craters with diameters ranging from 200 to 400 km which occurred between 3.85 to 3.6 billion years. Of course the size of these impact craters is partly related to the size of the impactors. Other factors such as density, velocity, and the angle of the impactor, as wells as characteristics of the target, will also play roles^86,87^.

With these considerations, four impactors are likely to have triggered the warming episode that initiated the floods discussed in our investigation; the one that caused the Antoniadi crater (3.83, 4.01, 21.4, 60.8), the one that formed the Vinogradov crater (3.76, 210, − 19.8, − 37.7), the one that produced the Lowell crater (3.72, 199, − 50.0, − 81.4), and the one that created the Galle crater (3.61, 223, − 50.6, − 30.9). Data in the parentheses are from^34^ and include (from left to right): the age of the crater in billions of years before present based on Hartman’s isochron, crater diameter in kilometer, latitude, and longitude, respectively. Future refinement in age dating should resolve this issue.

## Conclusions

Our study suggests that the SU and the HPU were deposited by gigantic flash floods after the emergence of Mt. Sharp when Gale crater had acquired its modern morphology. The flood formed giant gravel antidunes of the HPU which were at least 10 m high and 150 m apart. The SU deposited when floods waned eroding ridge crests and depositing sediments on the up-flow sides of antidunes of the underlying the HPU. Each patch is concave upward, consists of 5–10 m of south dipping layers, and is 50–200 m long and wide. At the Kimberley region, flood waters were at least 24 m deep and had a velocity of 10 m/s. The most likely cause of flooding was melting of ice by heat generated by a large impact. The event also mobilized CO_2_ and CH_4_ from their solid reservoirs that combined with H_2_O vapor to form a short period of warm and wet climate. Condensation of water vapor produced torrential rain planetwide some of which entered Gale crater and combined with water coming down from Mt. Sharp to produce gigantic flash floods that deposited the HPU and the SU.

## Materials and methods

The study was conducted by analysis of images taken by Mars Hand Lens Imager (MAHLI) and Mastcam cameras on board the Curiosity rover. MAHLI is a 2-megapixel color camera with a focusable macro lens that is mounted on Curiosity’s robotic arm to investigate rocks and minerals in Gale crater^88^. It acquires in-focus images at working distances ranging from 2.1 cm to infinity. It is capable of resolving fine sand grains^89^. Mastcam cameras are a multispectral imaging system, which consist of two digital cameras mounted on the rover’s mast (1.97 m above the ground). The left and right cameras have 34 mm (M-34) and 100 mm (M-100) focal lengths respectively, yielding pixel scales of 0.22 and 0.074 mrad/pixel, respectively. Mastcams are capable of full color panoramic and stereoscopic measurements^89^. Mastcams images were used to delineate sedimentary structures, sedimentary facies, stratal bounding surfaces and sedimentary architecture and determine dip directions of bedding. MAHLI and Mastcam images were used for sedimentological and textural characterization of targets. Criteria for identification of targets as sedimentary rocks include the presence of rounded grains, sedimentary structures, and layering. Then, dominant grain size was used to classify targets as mudstones or sandstones.

## Supplementary information

Supplementary Information 1.

Supplementary Information 2.

Supplementary Information 3.

Supplementary Information 4.

Supplementary Information 5.

Supplementary Information 6.

Supplementary Information 7.

Supplementary Information 8.

Supplementary Information 9.
