# Supplementary figures and images for: Deposits from giant floods in Gale crater and their implications for the climate of early Mars
Source: Sci Rep. 2020 Nov 5;10:19099. doi: 10.1038/s41598-020-75665-7 (PMC7645609; doi:10.1038/s41598-020-75665-7)

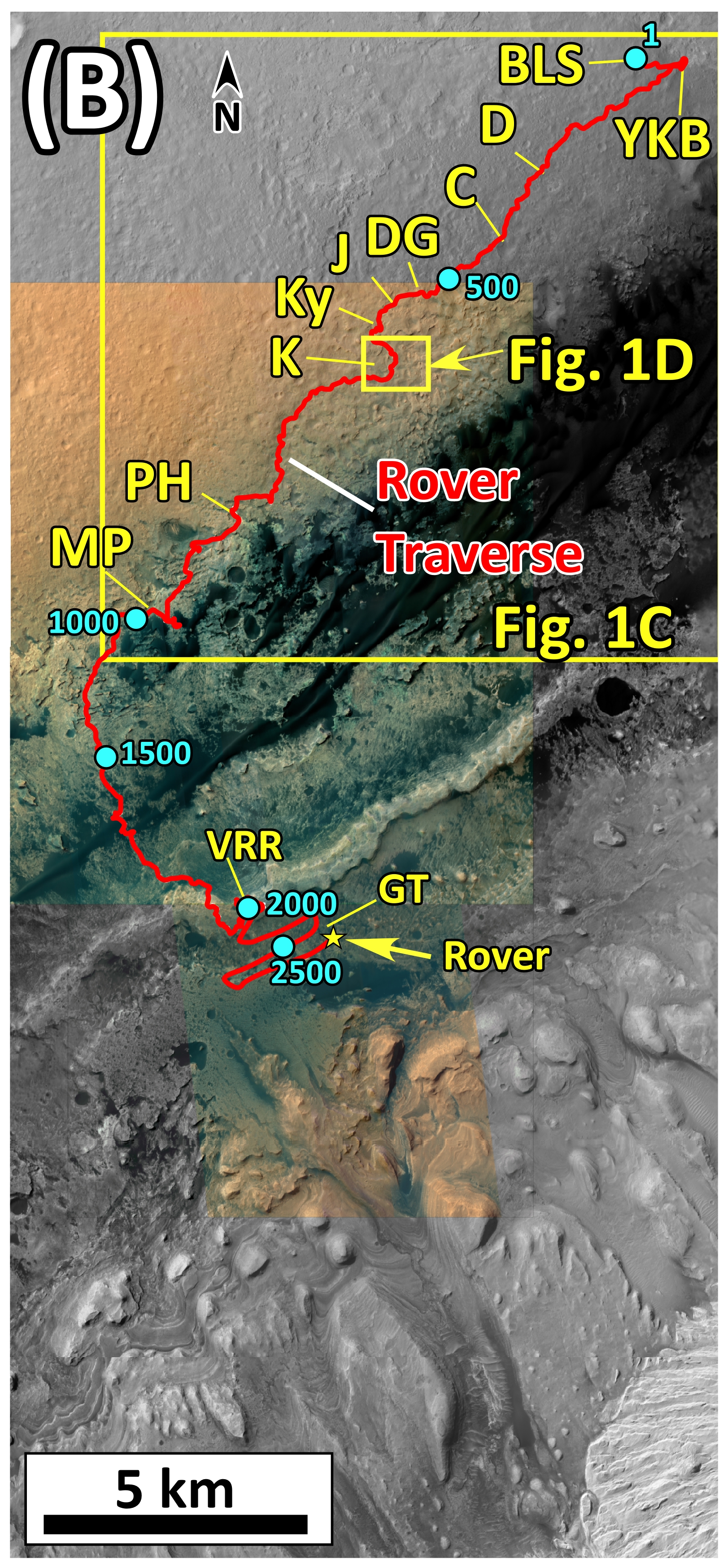

Supplement: Supplementary file 1 — Supplementary Information 1. [file 41598_2020_75665_MOESM1_ESM.jpg]

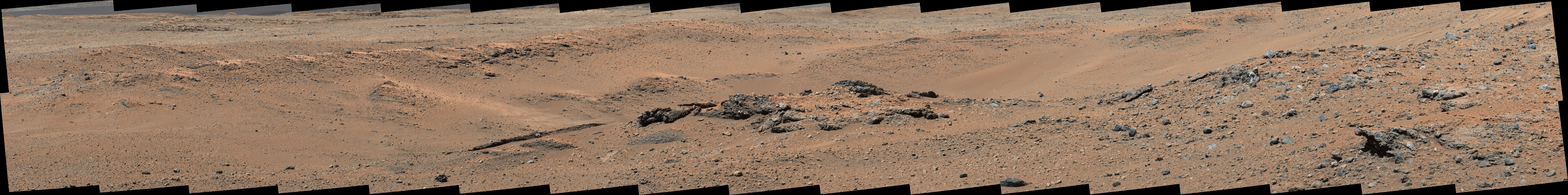

Supplement: Supplementary file 2 — Supplementary Information 2. [file 41598_2020_75665_MOESM2_ESM.png]

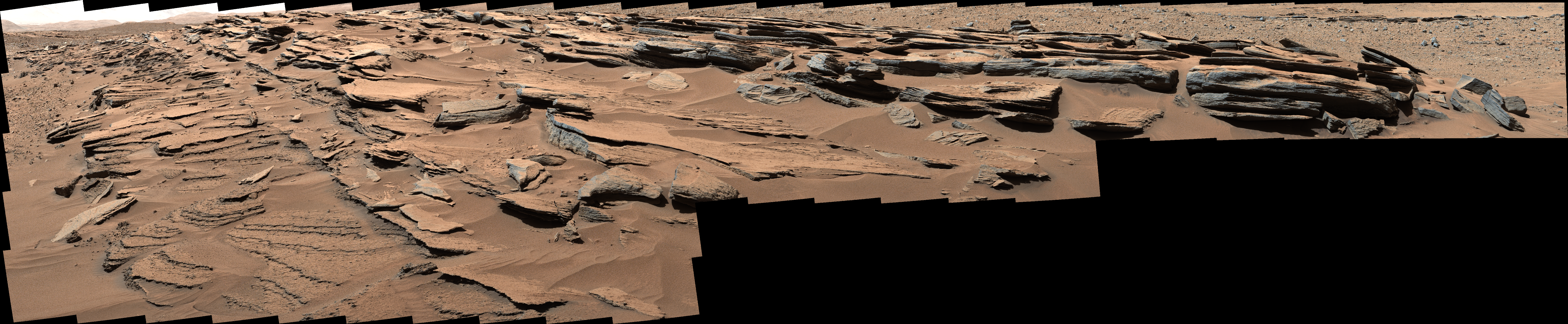

Supplement: Supplementary file 3 — Supplementary Information 3. [file 41598_2020_75665_MOESM3_ESM.png]

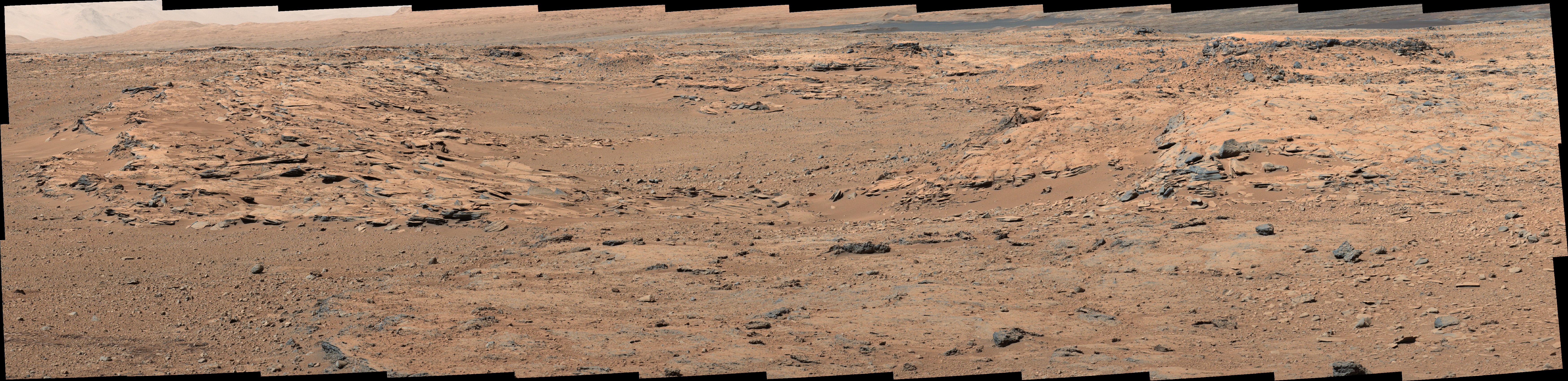

Supplement: Supplementary file 4 — Supplementary Information 4. [file 41598_2020_75665_MOESM4_ESM.png]

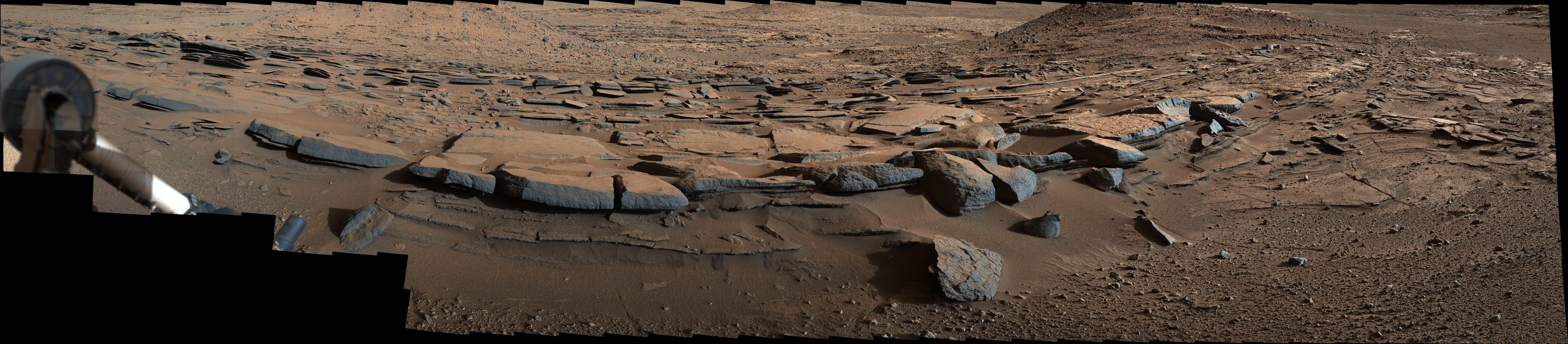

Supplement: Supplementary file 5 — Supplementary Information 5. [file 41598_2020_75665_MOESM5_ESM.png]

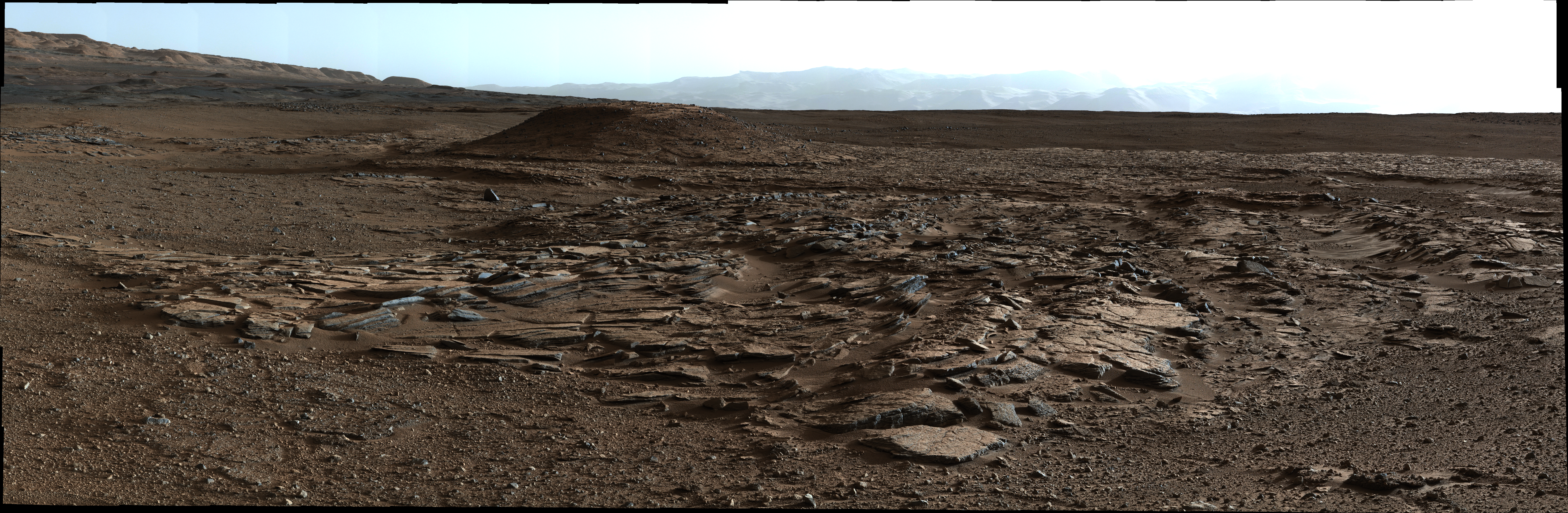

Supplement: Supplementary file 6 — Supplementary Information 6. [file 41598_2020_75665_MOESM6_ESM.png]

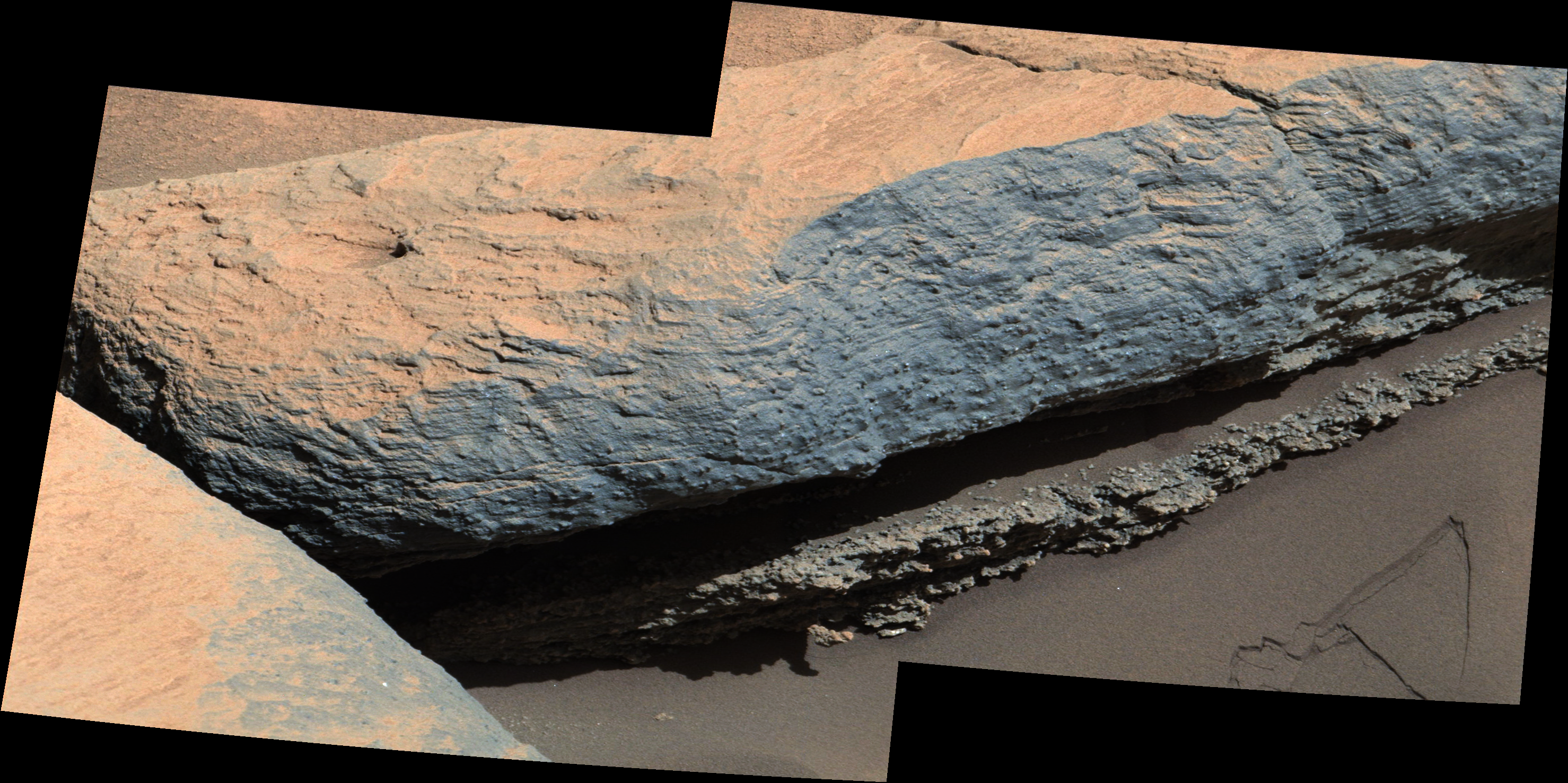

Supplement: Supplementary file 7 — Supplementary Information 7. [file 41598_2020_75665_MOESM7_ESM.png]

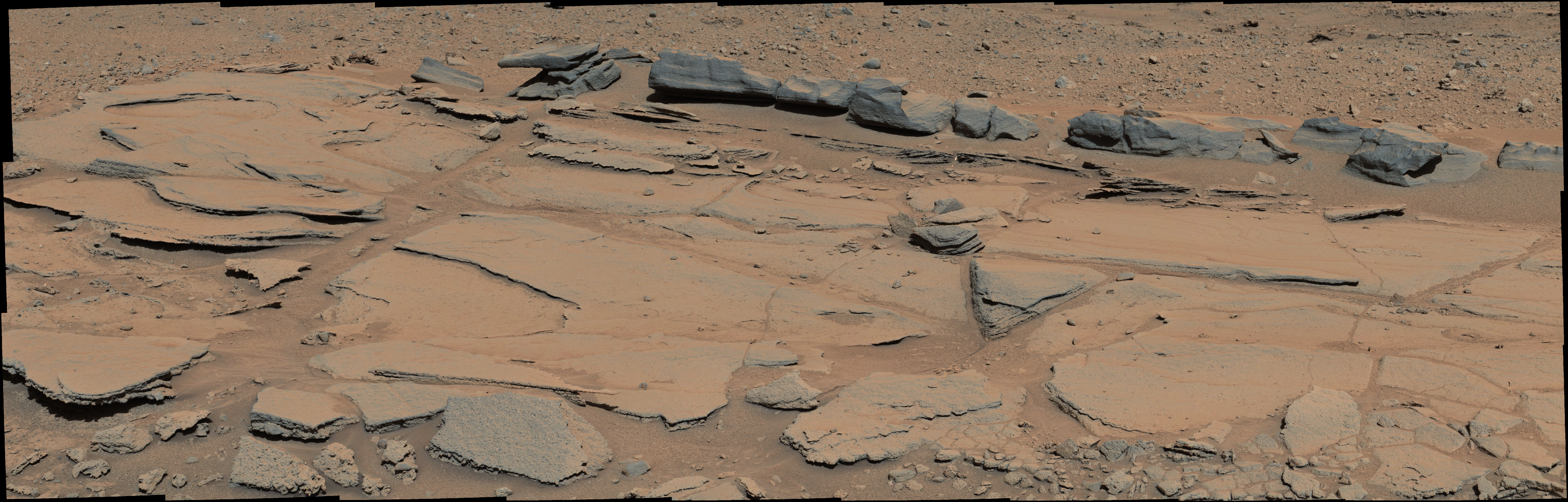

Supplement: Supplementary file 8 — Supplementary Information 8. [file 41598_2020_75665_MOESM8_ESM.png]
