# Supplementary material for: Deposits from giant floods in Gale crater and their implications for the climate of early Mars
Source: Sci Rep. 2020 Nov 5;10:19099. doi: 10.1038/s41598-020-75665-7 (PMC7645609; doi:10.1038/s41598-020-75665-7)
Supplement: Supplementary file 9 — Supplementary Information 9. [file 41598_2020_75665_MOESM9_ESM.pdf]

Supplemental Figure 9

Deposits from Giant Floods in Gale Crater and Their Implications for the Climate of Early Mars

E. Heydari (1)*, J. F. Schroeder (2), F. J. Calef (2), J. Van Beek (2), S. K. Rowland (3), and T. J. Parker (2), A. G. Fairén (4)

Supplementary Figure 9:

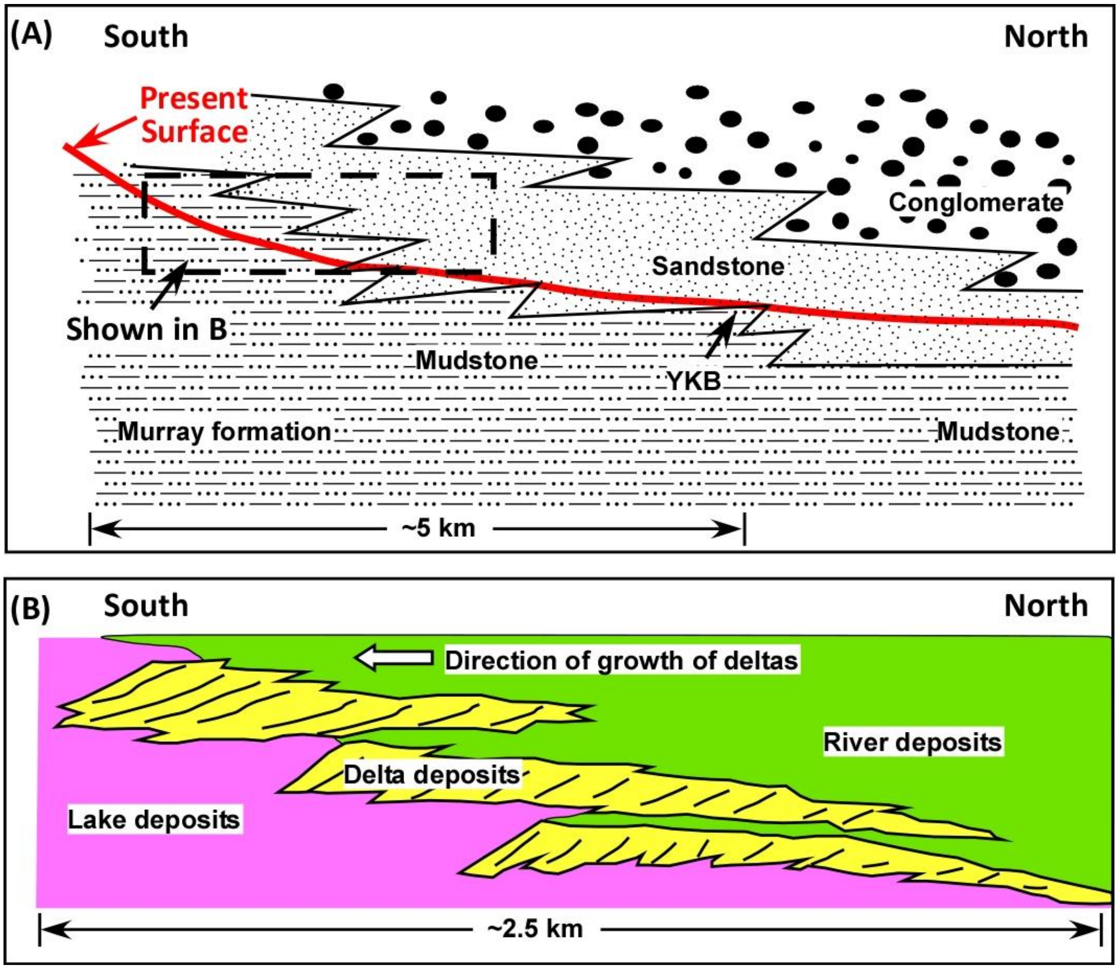

Supplemental Figure 9. Schematic drawing shows the existing fluvial-deltaic-lacustrine interpretation of strata examined by the Curiosity rover (modified from Grotzinger et al., 2015). The proposed model involved four rock units: the Hummocky Plains Unit (HPU), the Striated Unit (SU), the Rugged Terrain Unit (RTU), and the Murray formation (MF). **(A)** Schematic drawing represents north to south facies transition from conglomerate to mudstone exposed along the path of the Curiosity rover. These lithofacies were

considered time equivalent, that is, formed side-by-side at the same time. They migrated southward through time. These authors concluded all four rock units (the HPU, the SU, the RTU, and the MF) deposited when Gale crater was initially being filled. After the lake dried up, these rock units were buried by 5 km of sediments that filled the crater. Subsequent erosion at the margins of Gale crater resulted in the emergence of Mt. Sharp. It also formed the present-day exposure pattern of strata examined by the Curiosity rover. This erosion surface is marked by a thick red line. According to this scenario, all four rock units examined by the Curiosity rover pre-dated the emergence of Mt. Sharp. YKB: Yellowknife Bay. **(B)** Schematic diagram shows proposed depositional environments of lithofacies shown in A. It suggests alluvially-deposited sediments on the northern areas of the rover path transitioned southward into fluvial sandstone, then to deltaic sandstone, and finally to lacustrine mudstone. In this interpretation, The Striated Unit formed a series of small deltas that migrated southward due to rises in lake level. The original Figure was in black and white. For clarity, we colored the lithofacies to match those in Figure 1C of the manuscript.

Reference

Grotzinger, J. P. et al. 215. Deposition, exhumation, and paleoclimate of an ancient lake deposit, Gale crater, Mars. Science, **350;** doi:10.1126/science.aac7575 (2015).
